# South West Urological Club

**Published:** 1988-11

**Authors:** 


### South West Urological Club

### Meeting at Torbay, Summer 1987

### PRESENTATION AND MANAGEMENT OF THE BENT PENIS

**K. M. Desai and J. C. Gingell**
Southmead Hospital, Bristol

Curvature of the erect penis is not infrequently seen in urological practice. Forty-five men (age range 16–68 yrs; mean 49 yrs) were referred to our department with this complaint between August 1985 and February 1987 of whom 43 were associated with Peyronie's disease and two were "congenital". Significant medical conditions co-existed in 16 (37%) of the patients with Peyronie's disease, consisting of diabetes, Dupuytren's contracture, psoriasis and ulcerative colitis. Possible aetiological factors were identified in only 35%, comprising of i) trauma to the erect penis during intercouse (16%), ii) blunt injuries to the flaccid penis and perineum (10%), iii) urinary tract infection (9%). Apart from curvature, additional presenting symptoms included a noticeable thickening along the penis (88%), pain on erection (44%), reduced glanular sensation (21%) and decreased turgidity of the distal shaft or glans (19%). Erectile rigidity was described as normal in 51%, reduced but "adequate" for penetration in 30%, and poor in the remaining 19%. Intercourse was reported to be difficult or impossible by 70% of the men, a further 9% having stopped attempting it because of painful erections.

The majority of patients were evaluated by means of papaverine induced erections in the outpatient clinic. This not only allowed satisfactory photographic documentation of the type and degree of curvature (dorsal or dorso-lateral in 55%, ventral or ventro-lateral in 16%, left lateral in 16%, non-significant in 13%) by using a Polaroid AF 700 camera, but also a better delineation of the plaque and/or pathological fibrosis within the corpora was achieved by palpating the tumescent penis. A simultaneous dynamic Doppler study of the penile arteries was performed to exclude underlying arterial insufficiency.

Conservative treatment was recommended in 67% of the patients with Peyronie's disease, in most of whom the condition had either not stabilised or was causing minimal deformity. Ten patients (22%) underwent a Nesbit's procedure, semi-rigid penile prostheses being implanted in 6 men (14%) who had associated loss of erectile rigidity considered to be organic in aetiology; of the patients treated conservatively, 28% have subsequently reported resolution of symptoms and ability to have intercourse.

### PEYRONIE'S DISEASE
### The results of conservative treatment

**Mr M. M. Kirollos and Mr R. A. Bradbrook**
Torbay Hospital, Torquay

Controversy still exists about the management of Peyronie's disease two centuries after its original description. Spontaneous regression of the disease makes evaluation of the results of any treatment difficult and unreliable.

In this on-going study a 'no-interference' policy was adopted in an attempt to document accurately the natural history of the disease and set up the "controls" needed for assessing the results of any therapy.

Eleven patients were followed up for an average period of 18 months. The Peyronie's plaque – averaging 3×2 cm in size – was dorsal in 8 patients and ventral in 3 patients. During the period of the study the plaque disappeared or dramatically diminished in 6 patients and moderately decreased in 3 patients. The angulation on erection disappeared or improved dramatically in 4 patients and moderately in 4 patients. Four patients found it impossible to have sexual intercouse of which 2 became sexually active later. Five patients experienced difficulties on intercourse, 3 of them achieved significant improvement.

In general, 50–80% of the patients improved spontaneously. Firstly pain on erection subsides followed by the resolution of the plaque and subsequently an improvement in function. No interference for 2 years after presentation is a policy to be recommended.

### PRACTICAL PROBLEMS WITH UROSTOMY

**D. J. Chadwick and M. J. Stower**
Bristol Royal Infirmary, Bristol

Twenty-one urostomists have been interviewed and examined to determine the effect of the urostomy on their life-style. These included 11 men and 10 women with a mean age of 51. The indications for urinary diversion included carcinoma of bladder (6), neuropathic bladder (8), other benign urological disease (6) and vesico-colic fistula.

Nineteen patients stated that their quality of life since operation had improved. All except 3 patients were able to return to work or continue with normal activites, but 8 had been forced to modify their social activities in some way. Other problems included sexual relations and the wearing of seat belts.

The most common problem with the urostomy was leakage, which was generally infrequent and usually occurred beneath the base plate. Urine leakage was unrelated to stoma length or the patient's physique, but the site of the stoma and the presence of para-stomal hernia appeared to be important. Minor skin problems were common, although difficulties with bleeding, encrustation, odour and mucus were unusual. The patients considered the stomatherapist to be vitally important.

Although problems with urostomies exist, they are usually minor and do not interfere with patient's way of life to any great degree.

### UROLOGIST OPERATED OUTPATIENT ULTRASOUND SCANNING – A COST EFFECTIVE TOOL

**S. G. Vesey, G. N. Lumb and P. J. O'Boyle**
Musgrove Park Hospital, Taunton

A urologist operated outpatient clinic ultrasound scanning service was assessed over the course of 28 urological clinics. A total of 679 patients attended during the study period. Ultrasound scanning was performed on 190 (28%) patients. Scanning was performed whenever the urologist felt it was indicated. The indications were bladder outflow symptoms (111), loin pain (21), haematuria (15), testicular pathology (13) and miscellaneous (30). Scanning accuracy was assessed by referring patients for formal X-ray department ultrasound. In all cases urologist ultrasound scan findings, both positive and negative, were shown to be accurate.

The routine provision of such an ultrasound service has significant financial merits. Patients scanned during clinic need not return for formal X-ray department scans and as a result need not return for subsequent clinic review. Patient transport costs could result be lessened. Clinic waiting lists could be reduced as fewer return appointments become necessary. X-ray department workload could be lessened as referrals lessen.

At current costs it was estimated that the average District General Hospital could save in excess of £100,000 per annum by the routine provision of such an outpatient clinic scanning facility.

### A UROLOGY DIAGNOSTIC INDEX

**D. A. Gillatt, M. J. Stower and Paul Abrams**
Southmead Hospital, Bristol

The ever increasing pressure for audit of clinical practice means there is an urgent need for efficient methods of collection and correlation of data. Unless clinicians undertake medical audit, non clinicians may well take the initiative.

Every district within the South West region now has a main frame computer installed. Within each hospital the central computer has links to terminals within various departments. Ultimately each unit will have access to the patient administration system, waiting lists, and pathology services.

A Urology Diagnostic Index has been set up within the patient administration system (PAS). Basic details such as hospital number, patient's address and general practitioner are automatically available from the PAS. Details on each urological admission and outpatient episode are fed into the diagnostic index. Twenty-four separate pieces of information can be stored for each episode. The system allows automatic generation of a discharge summary for each patient. It will also allow the prospective collection of data both for audit and research interests.

It is feasible that regional and sub-regional studies could be undertaken using this system. The Department of Urology at Southmead Hospital intends to use this system to initiate a bladder tumour registry.

### SERUM PROSTATE SPECIFIC ANTIGEN MEASUREMENT IN PROSTATIC DISEASE

**D. A. Gillat, M. A. Ferro, P. J. B. S. Smith and J. C. Gingell**
Bristol Royal Infirmary and Southmead Hospital, Bristol

Prostate specific antigen (PSA) is a glycoprotein with a molecular weight of approximately 35,000. It is present in the cytoplasm of prostatic cells only. It can now be assayed in serum using a radioimmunoassay.

Two hundred and fifty men have had serum PSA measurements. Thirty "normal " controls, had a mean PSA level of 2.18 ng/ml (1.9–4.7 ng/ml) one hundred and twenty with benign prostatic hyperplasia had a mean PSA of 13.0 ng/ml (1.9–80.2 ng/ml). Thirty-eight men with localized prostatic cancer had mean levels of 59.4 ng/ml (1.9–374) and sixty-two with metastatic disease 314 ng/ml (range 2.0–3574). Eighty-three men undergoing hormonal manipulation, either by orchidectomy, LH–RH analogues or antiandrogens, have been studied longitudinally. Four patients showed no clinical response and in each case PSA levels continued to rise. In the remaining patients a clinical response was paralleled by a fall in serum PSA levels. Disease progression has occurred in 20 men, in 19 (95%) progression was accompanied by an increasing serum PSA.

Overall for metastatic disease PSA had a sensitivity of 95%, compared with 60% for prostatic acid phosphatase and 85% for alkaline phosphatase.

It is concluded that serum PSA measurement is a sensitive marker for advanced prostatic cancer and useful in monitoring its treatment.

### WHY ARE THERE STILL DELAYS IN DIAGNOSING BLADDER CANCER? A PRELIMINARY STUDY

**M. J. Stower**
Bristol Royal Infirmary and Southmead Hospital, Bristol

To date seventy patients with a newly diagnosed bladder cancer (49 males, 21 females) with a mean age of 67.2 (range 29–93) yr have been prospectively interviewed and the notes reviewed to ascertain where if any delay had occurred in making the diagnosis.

Painless haematuria was the presenting symptom in 44 patients.

The median total delay in making the diagnosis from the first symptom was 18 (1–323) weeks. The median patient delay in seeking medical advice was 1 (1–263) week; the delay before the GP referred the patient to hospital was 1 (1–308) week, whilst the delay that occurred in the hospital service was 7 (1–226) weeks. The longest delay was seen in patients under 50 yr old. Patients with painful haematuria or "cystitis" symptoms had a delay 6 weeks longer than those with just painless haematuria, which was seen in both GP and hospital service. Hospital delay was due equally to waiting for out-patient clinic appointments and waiting for admission.

Of particular concern is that this study shows that most of the delay in diagnosing bladder cancer is due to the hospital service.

